# Influence of distillers grains with solubles on bull growth and reproductive traits^1^

**DOI:** 10.1093/tas/txz189

**Published:** 2020-01-11

**Authors:** Parker A Henley, Giorgia Podico, Edgar Garrett, Claire Kaplan, William T Meteer, Joshua C McCann, Igor Canisso, Daniel W Shike

**Affiliations:** 1 Department of Animal Sciences, University of Illinois at Urbana-Champaign, Urbana; 2 Department of Veterinary Clinical Medicine, College of Veterinary Medicine, University of Illinois, Urbana IL; 3 University of Illinois Extension, Orr Research and Demonstration Center, Baylis

**Keywords:** bulls, cattle, distillers grains, fertility, reproduction

## Abstract

This study evaluated the effects of offering growing bulls a diet with 40% modified wet distillers grains plus solubles (MWDGS; dry matter [DM] basis) on growth, composition, hoof scores, and reproductive performance. Simmental × Angus bulls (*n* = 28) were stratified by body weight (BW; 316 ± 29 kg), sire, and day 0 semen production (Y/N) and assigned into one of six pens. Pens were randomly assigned to one of two dietary treatments (*n* = 3 pens/treatment): 1) offered free-choice access to a corn-based diet with no MWDGS (CON) or 2) offered free-choice access to a diet with 40% MWDGS (DST; DM basis). Bulls were offered treatments for 140 d and, then, switched to a free-choice, common, low-energy diet for an additional 70 d. Bull BW, body condition score (BCS), hip height (HH), ultrasound (rump fat depth [RF], 12th rib fat thickness [BF], marbling score [MS], and longissimus muscle depth [MD]), hoof evaluations, breeding soundness examination (BSE), and semen evaluations were performed on days 0, 28, 56, 84, 112, 140, 175, and 210. There was a tendency (*P* < 0.09) for a treatment × time effect for BW. Bulls fed DST tended (*P* ≤ 0.10) to be heavier on days 28 and 56 than CON bulls. A treatment × time effect was detected (*P* < 0.01) for RF. Bulls fed DST had greater (*P* = 0.02) RF on day 84 than CON bulls. Treatment × time and treatment effects were detected (*P* ≤ 0.05) for BF, in which DST bulls had greater (*P ≤ 0*.05) BF on days 84, 112, and 175 and tended (*P* ≤ 0.10) to be greater on days 56 and 210 than CON bulls. A treatment × time effect was detected (*P* < 0.02) for percentage of major sperm defects. Bulls fed DST had a greater (*P* < 0.01) percentage of major sperm defects on day 140 than control bulls. There was a tendency (*P* = 0.09) for a treatment × time effect for percentage of sperm with proximal droplets. Bulls fed DST had a greater (*P* = 0.01) percentage of sperm with proximal droplets on day 140 than CON bulls. In conclusion, offering growing bulls a diet with 40% MWDGS (DM basis) resulted in heavier BW at days 28 and 56, increased RF at day 84, and increased BF and increases in both major sperm defects and sperm with proximal droplets at day 140. However, after 70 d on the common low-energy diet, there were no carryover effects for any growth, composition, hoof, or reproductive measures except for a tendency for BF.

## INTRODUCTION

The two most important factors that drive the financial success of a cow–calf enterprise are nutrition and reproduction (Hess et al., 2005). Additionally, Bellows et al. (2002) reported that reproductive conditions and diseases could cost beef cattle producers as much as $502 million in lost income yearly. A significant portion of the reproductive failures in cow–calf enterprises are due to the fertility of the herd bull (Flowers, 2013). A majority of spring-calving seedstock operations in the Midwest market their bulls in the early spring, but producers, generally, do not expose them to cows until late spring or early summer. Many seedstock producers develop bulls on grain-based, high-energy diets prior to sale. Costs associated with feeding these diets have led producers to consider cheaper alternatives, such as distillers grains with solubles. However, distillers grains with solubles can contain relatively high concentrations of S and crude fat, and although variability in new generation ethanol refineries has been decreased, excess crude protein (CP), crude fat, and S in the diet may be a concern. Van Emon et al. (2013) reported a linear decrease in spermatozoa concentration of rams as distillers dried grains with solubles (DDGS) increased in the diet (0%, 15%, and 30%; dry matter [DM] basis). Additionally, Crane et al. (2018) fed increasing concentrations (0%, 15%, 30%, and 45%; DM basis) of DDGS and observed decreased scrotal circumference but increased spermatozoa concentration and morphologically normal sperm of rams. Due to minimal research with inconsistent results, it is not clear if distillers grains with solubles should be included in the diet of breeding bulls; thus, further research is warranted. Therefore, the objectives of this study were to evaluate the effects of offering growing bulls a diet with 40% modified wet distillers grains plus solubles (MWDGS; DM basis) on body weight (BW), body condition score (BCS), hip height (HH), ultrasound (rump fat depth [RF], 12th rib fat thickness [BF], marbling score [MS], and longissimus muscle depth [MD]), hoof evaluations, reproductive measures, percentage of bulls that were pubertal, percentage of bulls with a satisfactory breeding soundness examination (BSE), and semen motility, morphology, and concentration. The authors hypothesized that offering a diet with 40% MWDGS (DM basis) would negatively affect semen motility, morphology, and concentration at the end of the treatment period. However, bull growth performance, body composition, and hoof evaluations would not be affected.

## MATERIALS AND METHODS

All experimental procedures were approved by the Institutional Animal Care and Use Committee of the University of Illinois (IACUC #17240) and followed the guidelines recommended in the Guide for the Care and Use of Agricultural Animal in Agricultural Research and Teaching (FASS, 2010). The experiment was performed over a 210-d period from October 2017 to May 2018.

### Animals and Experimental Design

Spring-born, Simmental × Angus bulls (initial BW = 316 ± 29 kg, initial age = 240 ± 6 d, *n* = 28) housed at the University of Illinois Beef Cattle and Sheep Field Laboratory in Urbana, IL, were utilized to evaluate the effects of a diet with 40% MWDGS (DM basis) on bull reproductive and growth performance. Bulls were stratified by BW, sire, and if they produced semen on day 0 (*n* = 7) and assigned into one of six pens with four or five bulls per pen. Bulls were housed in a barn constructed of wood frames with ribbed metal roofs and siding on the north, west, and east sides. The south side of each barn was covered with polyvinyl chloride-coated 1.27-by-1.27-cm wire mesh bird screen and equipped with retractable curtains for wind protection. Within the barn, pens (4.88 × 9.76 m) had concrete slats covered with rubber mats. Pens were randomly assigned to one of two dietary treatments (*n* = 3 pens/treatment; Table 1): 1) offered free-choice access to a corn-based diet with no MWDGS (CON) or 2) offered free-choice access to a diet with 40% MWDGS (DST; DM basis). Bulls were offered treatments for 140 d and, then, switched to a free-choice, common, low-energy diet for an additional 70 d. Forty days before the start of the study, bulls were weaned and shipped from Orr Agricultural Research and Demonstration Center in Baylis, IL, to the Beef and Sheep Field Research Laboratory in Urbana, IL, where they were offered free-choice access to a common diet (50% alfalfa haylage, 45% corn silage, and 5% ground corn-based supplement; DM basis) and adapted to the GrowSafe automated feeding system (model 4000E, GrowSafe Systems Ltd., Airdrie, AB, Canada). Fifty-two days prior to weaning, bulls received 5-mL Bovi-shield Gold FP5 VL5 HB (Zoetis, Parsippany, NJ), 2-mL One Shot Ultra 8 (Zoetis, Parsippany, NJ), and 2-mL MpB Guard (American Health Inc., Ronkonkoma, NY). Twenty-one days prior to weaning, bulls received 5-mL Bovi-shield Gold FP5 VL5 HB (Zoetis, Parsippany, NJ), 2-mL Ultra Choice 8 (Zoetis, Parsippany, NJ), 2-mL MpB Guard (American Health Inc., Ronkonkoma, NY), and 1-mL/nostril Inforce 3 (Zoetis, Parsippany, NJ). At the time of weaning, all bulls received 1-mL/9.98-kg BW Eprinex (Merial, Duluth, GA). One bull from DST had to be removed from the experiment for chronic respiratory disease.

**Table 1.** Ingredients of diets fed to growing bulls during treatment period and following common low-energy period

	CON transition steps^1^					DST transition steps					
Item	1	2	3	4	5	1	2	3	4	5	Low energy
Ingredient inclusion, % DM											
Dry-rolled corn	0	10	20	30	40	–	–	–	–	–	–
MWDGS^2^	–	–	–	–	–	0	10	20	30	40	–
Chopped grass hay	–	–	–	–	–	–	–	–	–	–	60
Alfalfa haylage	50	50	40	30	25	50	50	40	30	25	–
Soybean hulls	0	0	5	15	25	0	0	5	15	25	10
Corn silage	45	35	25	15	0	45	35	25	15	0	20
Supplement^3^											
Ground corn	4.37	4.37	5.41	5.41	5.41	4.37	4.37	8.22	8.22	8.22	7.62
Limestone	0.45	0.45	0.90	0.90	0.90	0.45	0.45	1.59	1.59	1.59	1.59
Urea	0.67	0.67	–	–	–	0.67	0.67	–	–	–	0.60
Blood	–	–	3.50	3.50	3.50	–	–	–	–	–	–
Trace mineral premix^4^	0.089	0.089	0.091	0.091	0.091	0.089	0.089	0.091	0.091	0.091	0.91
Rumensin 90	0.015	0.015	0.015	0.015	0.015	0.015	0.015	0.015	0.015	0.015	0.016
Tylosin^5^	0.010	0.010	0.010	0.010	0.010	0.010	0.010	0.010	0.010	0.010	0.010
Fat	0.076	0.076	0.075	0.075	0.075	0.076	0.076	0.075	0.075	0.075	0.075

^1^Treatments were as follows (*n* = 3 pens/treatment): 1) offered free-choice access to a corn-based diet with no modified wet distillers grains plus solubles (MWDGS; CON) or 2) offered free-choice access to a diet with 40% MWDGS (DST; DM basis). Diets were fed to bulls (initial BW = 316 ± 29 kg, initial age = 240 ± 6 d) as follows: step 1 = days −35 to 1; step 2 = days 1 to 7; step 3 = days 8 to 14; step 4 = days 15 to 21; step 5 = days 22 to 140; low energy = days 141 to 210.

^2^Modified wet distillers grains plus solubles.

^3^Premix.

^4^8.5% Ca, 5% Mg, 7.6% K, 6.7% Cl, 10% S, 0.5% Cu, 2% Fe, 3% Mn, 3% Zn, 278 mg/kg Co, 250 mg/kg I, 150 mg/kg Se, 2,205 KIU/kg Vit A, 662.5 KIU/kg Vit D, 22,047.5 IU/kg Vit E.

^5^Tylan 40, Elanco Animal Health, Greenfield, IN.

### Sample Collection and Analytical Procedures

Full BW were collected on two consecutive days prior to feeding (approximately 0700 h) at the beginning of the treatment period (days 0 and 1) and at the end of the treatment period (days 139 and 140). Additional full BW were collected prior to feeding (approximately 0700 h) on days 28, 56, 84, 112, 175, and 210. All BW were taken using a Flying W squeeze chute (Flying W Livestock, Watonga, OK) equipped with a Tru-Test (Tru-Test Inc., Mineral Wells, TX) weighing system. On days 0, 28, 56, 84, 112, 140, 175, and 210, HH was evaluated using a measuring stick (Altitude Stick, Nasco, Fort Atkinson, WI). Individual feed intake was collected using the GrowSafe automated feeding system (model 4000E, GrowSafe Systems Ltd., Airdrie, AB, Canada) throughout the entire study.

Bull RF, BF, MS, and MD were estimated on days 0, 28, 56, 84, 112, 140, 175, and 210 via ultrasound. Ultrasound measurements were taken by trained personnel using an Aloka 500SV (Wallingford, CT) B-110 mode instrument equipped with a 3.5-Mhz general-purpose transducer array. Back fat depth, MS, and MD were taken in transverse orientation between the 12th and 13th ribs approximately 10 cm distal from the midline. Images were analyzed using CPEC imaging software (Cattle Performance Enhancement Company LLC., Oakley, KS).

Hoof evaluations were quantified using the American Angus Association’s simple foot scoring system, which characterizes cattle for two traits: foot angle and claw set. Both scores are ranked on a 1–9 scoring system with 5 being ideal. An animal characterized as a 5 for foot angle would have a 45° angle to the pastern. Animals that are extremely straight in their front end and up on their toes would score a 1, while an animal with a very shallow heel and extremely long toes would score a 9. Animals must have symmetrical claws with some space between them in order for an animal to be scored with an ideal claw set or a 5. Animals scoring a 9 would have extreme scissor claw or screw claw with the curling and crossing of both claws, while animals scoring a 1 for claw set would have extremely weak, open or divergent claws

Feed ingredient samples were collected once every 2 wk throughout the experiment. Equal portions of each ingredient in each period were composited. Composite samples were dried and ground through a Wiley mill (1-mm screen, Arthur H. Thomas, Philadelphia, PA). Ingredients were analyzed for DM (24 h at 103 °C), neutral detergent fiber (NDF), and acid detergent fiber (ADF; using Ankom Technology methods 5 and 6, respectively; Ankom200 Fiber Analyzer, Ankom Technology), CP (Leco TruMac, LECO Corporation, St. Joseph, MI), ether extract (EE; Ankom method 2; Ankom Technology), and ash (600 °C for 2 h; Thermolyte muffle oven Model F30420C; Thermo Scientific, Waltham, MA). Additionally, samples were sent to Michigan State University Veterinary Diagnostic Laboratory (Lansing, MI) and concentrations of Ca, Cu, Mn, P, S, and Zn were analyzed using a Varian Vista-Pro Inductively Coupled Plasma Optical Emission Spectrometry (ICP/OES; Agilent Technologies Inc., Palo Alto, CA).

### Reproductive Performance

A complete BSE was performed on days 0, 28, 56, 84, 112, 140, 175, and 210. The examination included a subjective assessment of physical soundness, including evaluation of the legs, feet, and eyes. Each bull was assigned a BCS of 1–9, scrotal circumference (SC) was measured, and spermatic cord circumference (SCORD) was measured with a steel tape (Lane Manufacturing, Denver, CO) in centimeters at the widest portion of the scrotum or spermatic cord, respectively. Palpation of testicles and epididymides was performed to assess tone, symmetry, and presence of adhesions, scars, or malformations of external genitalia. Transrectal palpation was performed to assess the tone and symmetry of the accessory sex glands. During semen collection, the penis was examined for abnormalities. Bulls failed BSE if their semen sample exhibited <30% total motility, <70% normal sperm morphology, or an SC lower than recommended for their respective age range (Koziol and Armstrong, 2018).

Semen samples were collected on days 0, 28, 56, 84, 112, 140, 175, and 210. Sample collection occurred at approximately the same time in the morning, 0700–1100 h, to minimize the effects of daily hormonal fluctuations. To obtain semen samples, a 60‐mm upright weighted bull probe (Lane Manufacturing, Inc., Denver, CO) was placed rectally, and the programmed cycle on the electroejaculator (Pulsator IV, Lane Manufacturing, Inc., Denver, CO) was allowed to run. Once at least 3 mL of semen was collected, the electroejaculation was concluded and the probe was removed. If an appropriate ejaculate was not obtained following the cycle, the bull was recorded as no semen was produced. Semen was collected into 15‐mL conical tubes using collecting handles and sleeves. Semen samples were transported in warm water (37 °C) bath to the laboratory for no longer than 15 m postcollection. Semen was diluted 1:100 in buffered formalin (10%), and 10 μL of diluted semen was placed into a hemocytometer chamber to assess the concentration (Improved Neubauer, Hausser Scientific, Horsham, PA). The concentration (n. sperm/mL) was calculated by multiplying the dilution factor to the mean number of sperms counted in five squares to a fixed factor (10,000). Computer‐Assisted Sperm Analysis (CASA) equipment (Spermvision, MiniTube of America, Inc., Verona, WI) was used to obtain overall and progressive motility by averaging seven readings from various portions of the slide or thousand cells. As per the manufacturer’s defaults, CASA‐established set‐up parameters for bovine sperm were as follows: frame capture speed rate, 60 Hz; cell size (min/max), 18/60 μm^2^; threshold straightness, 50%; velocity average path cutoff, 56 μm/s; and velocity straight line cutoff, 28 μm/s. Morphological abnormalities were assessed by a single‐blinded observer by examining high‐power images (100× with phase-contrast lens) of multiple sections of the chambered slide. Images were used to classify morphology as normal or abnormal. Abnormalities were further defined as major or minor defects by the same observer for all samples on all collection days based on the standards set forth by Koziol and Armstrong (2018). After the assessment of 200 sperm cells from each diluted semen sample, the percentages of normal, major, and minor abnormalities were calculated. On days 0, 28, 56, 84, 112, 140, 175, and 210, bulls were evaluated for pubertal status. Bulls were determined to have reached puberty when at least 50 million sperm with at least 10% progressive motility were present in the ejaculate according to Wolf et al. (1965).

### Statistical Analysis

Data were analyzed as a stratified randomized design using pen as the experimental unit in SAS 9.4 (SAS Inst. Inc., Cary, NC). BW, BCS, HH, RF, BF, MS, MD, hoof angle score, and hoof claw score were analyzed as repeated measures in the MIXED procedure of SAS 9.4 (SAS Inst. Inc., Cary, NC) with fixed effects of treatment, time, and the interaction of treatment and time. Additionally, the most appropriate expected progeny differences (EPD) estimate was used as a covariate to help account for inherent genetic differences. If no EPD was appropriate, sire was included as a covariate. Baseline values collected at the start of the study were included as a covariate. Pen(treatment) was included as a random effect. Bull(Pen) was the subject of the REPEATED statement. The heterogeneous autoregressive (1) covariance structure was selected based on Akaike information criterion and experimental design fit. Reproductive measurers, semen evaluation, and semen morphology were analyzed as repeated measures in the MIXED procedure of SAS 9.4 (SAS Inst. Inc., Cary, NC) with fixed effects of treatment, time, and the interaction of treatment and time. Sire was used as a covariate to help account for inherent genetic differences. Bull semen production on day 0 (yes or no) was included as a covariate. Bull(Pen) was the subject of the REPEATED statement. The autoregressive (1) covariance structure was selected based on Akaike information criterion and experimental design fit. Binomial data (Bull pubertal status and bulls with a satisfactory BSE) were converted to working variables (*x*) based on the proportion of successes within *n* groups (six total pens). The working variables were transformed using sin^−1^(*x*)^0.5^ as described by Humblot et al. (1991) because of nonnormality. When *x* = 0, the observation 0 was replaced with (1/4) (1/*n*) and, when *x* = 1, the observation was replaced with 1 − (1/4) (1/*n*) (Bartlett, 1947). Estimates (of *y*) were back-transformed using (sin[y])^2^. The residuals for sperm concentration were not normally distributed and were transformed using the BoxCox procedure of SAS 9.4 (SAS Inst. Inc., Cary, NC) using *X*^0.25^. For the percentage of morphologically normal sperm, plots of residual versus fitted values showed a reduction in variation as the percentage normal approached 100%. A log transformation was, therefore, applied (log_e_ [100 − % normal]). For sperm morphological abnormalities, plots of residuals versus fitted values showed the variation increasing with the mean. A log transformation was performed for all morphologically abnormal traits and sperm concentration before analysis to stabilize the variance. Least square means were back-transformed for ease of interpretation. The SLICE statement was used to separate least square means when the interaction of treatment and time was significant (*P* ≤ 0.05). Significance was declared at *P* ≤ 0.05 and tendencies were noted at 0.05 < *P* ≤ 0.10. Means reported in tables and figures are least square means.

## RESULTS

**Figure 1.**
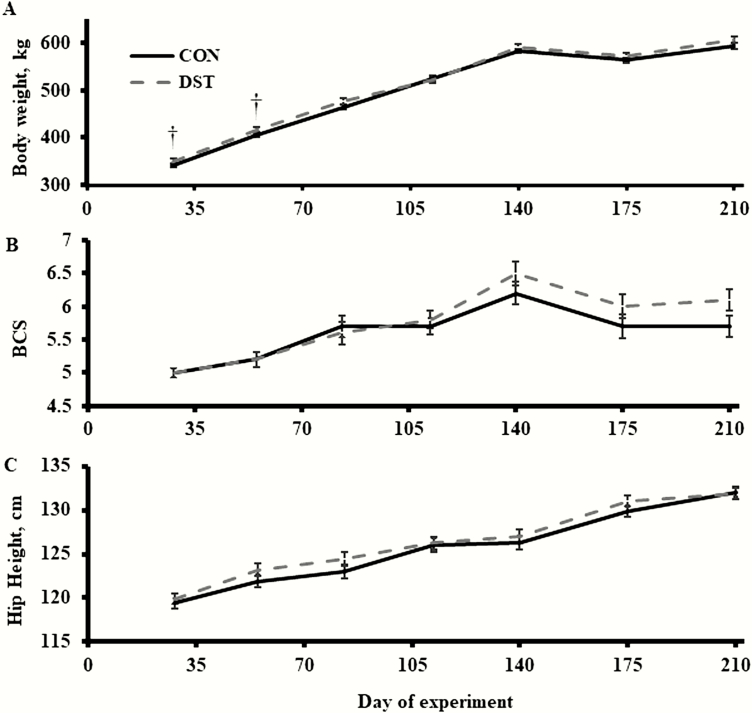
Effects of offering growing bulls a diet with 40% MWDGS (DM basis) on BW, BCS, and bull HH over time. Treatments were as follows (*n* = 3 pens/treatment): 1) offered free-choice access to a corn-based diet with no MWDGS (CON) or 2) offered free-choice access to a diet with 40% MWDGS (DST; DM basis). Bulls (initial BW = 316 ± 29 kg, initial age = 240 ± 6 d) were offered treatments for 140 d and, then, switched to a free-choice, common, low-energy diet for an additional 70 d. Significance of slice *P*-values are represented as: *P* ≤ 0.05 defined by *, and tendencies from 0.05 < *P* ≤ 0.10 are defined as †. Vertical bars represent the SEM. There was a tendency (*P* < 0.09) for a treatment × time effect for BW. Treatment × time and treatment effects were not detected (*P* ≥ 0.27) for BCS or HH. A time effect was detected (*P* ≤ 0.01) for BW, BCS, and HH.

There was a tendency (*P* < 0.09; Figure 1) for a treatment × time effect for BW. Bulls fed DST tended (*P* ≤ 0.10) to be heavier on days 28 and 56 than CON bulls. However, a treatment effect was not detected (*P* = 0.25) for BW. Treatment × time and treatment effects were not detected (*P* ≥ 0.27; Figure 1) for BCS or HH. Nevertheless, a time effect was detected (*P* ≤ 0.01) for BW, BCS, and HH, which BW and HH increased over time, whereas BCS increased to day 140 and, then, decreased to day 210. Treatment effects were not detected (*P* ≥ 0.17; Table 3) for days 1 to 140 average daily gain (ADG), days 140 to 210 ADG, days 1 to 140 dry matter intake (DMI), days 140 to 210 DMI, days 1 to 140 gain:feed (G:F), and days 140 to 210 G:F.

**Table 3.** Effects of offering growing bulls a diet with 40% MWDGS (DM basis) on average daily gain, dry matter intake, and gain:feed

	Treatment^1^			
Item	CON	DST	SEM	*P*-value
Average daily gain, kg/day				
days 1 to 140	1.93	1.95	0.05	0.70
days 140 to 210	0.14	0.23	0.10	0.61
Dry matter intake, kg/day				
days 1 to 140	11.1	10.6	0.40	0.44
days 140 to 210	9.5	9.4	0.24	0.69
Gain:feed, kg/kg				
days 1 to 140	0.17	0.18	0.005	0.17
days 140 to 210	0.02	0.03	0.011	0.63

^1^Treatments were as follows (*n* = 3 pens/treatment): 1) offered free-choice access to a corn-based diet with no MWDGS (CON) or 2) offered free-choice access to a diet with 40% MWDGS (DST; DM basis). Bulls (initial BW = 316 ± 29 kg, initial age = 240 ± 6 d) were offered treatments for 140 d and, then, switched to a free-choice, common, low-energy diet for an additional 70 d.

**Figure 2.**
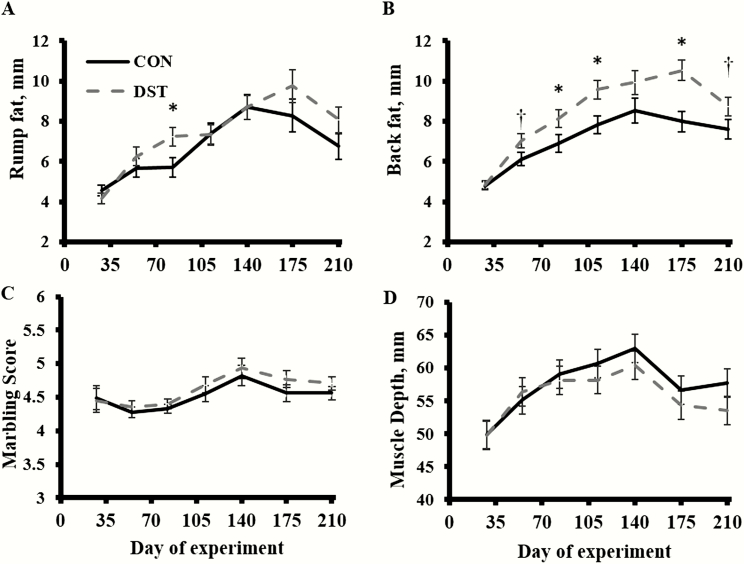
Effects of offering growing bulls a diet with 40% MWDGS (DM basis) on ultrasound parameters RF, 12th rib BF, MS, and loin MD over time. Treatments were as follows (*n* = 3 pens/treatment): 1) offered free-choice access to a corn-based diet with no MWDGS (CON) or 2) offered free-choice access to a diet with 40% MWDGS (DST; DM basis). Bulls (initial BW = 316 ± 29 kg, initial age = 240 ± 6 d) were offered treatments for 140 d and, then, switched to a free-choice, common, low-energy diet for an additional 70 d. Significance of slice *P*-values are represented as: *P* ≤ 0.05 defined by *, and tendencies from 0.05 < *P* ≤ 0.10 are defined as †. Vertical bars represent the SEM. A treatment × time effect was detected (*P *< 0.01) for RF. A treatment effect was not detected (*P* = 0.34) for RF. Treatment × time and treatment effects were detected (*P* ≤ 0.05) for BF. Treatment × time and treatment effects were not detected (*P* ≥ 0.16) for MS or MD. A time effect was detected (*P* ≤ 0.01) for RF, BF, MS, and MD.

A treatment × time effect was detected (*P* < 0.01; Figure 2) for RF. Bulls fed DST had greater (*P* = 0.02) RF on day 84 than CON bulls. However, a treatment effect was not detected (*P *= 0.34) for RF. Treatment × time and treatment effects were detected (*P* ≤ 0.05; Figure 2) for BF, in which DST bulls had greater (*P* ≤ 0.05) BF on days 84, 112, and 175 and tended (*P* ≤ 0.10) to be greater on days 56 and 210 than CON bulls. Bulls fed DST had greater (*P* = 0.05) BF than CON bulls. Treatment × time and treatment effects were not detected (*P* ≥ 0.16; Figure 2) for MS or MD. A time effect was detected (*P* ≤ 0.01) for RF, BF, MS, and MD, which bull RF, BF, MS, and MD increased to day 140 followed by decrease from days 140 to 210.

Treatment × time and treatment effects were not detected (*P* ≥ 0.55; Table 4) for foot angle or claw set. However, a time effect was detected (*P* ≤ 0.01) for foot angle and claw set. Foot angle initially decreased and then increased over time. Claw set increased over time.

**Table 4.** Effects of offering growing bulls a diet with 40% MWDGS (DM basis) on foot angle and claw set over time

	Treatment^1^			*P*-value^2^		
Item^3^	CON	DST	SEM	Trt	Time	Trt × Time
Foot angle				0.99	<0.01	0.91
day 28	5.6	5.7	0.22			
day 56	5.1	5.0	0.24			
day 84	5.5	5.5	0.24			
day 112	5.3	5.3	0.26			
day 140	5.2	5.2	0.23			
day 175	5.6	5.5	0.25			
day 210	5.9	5.9	0.28			
Claw set				0.73	<0.01	0.55
day 28	4.7	4.8	0.16			
day 56	4.9	4.9	0.20			
day 84	5.4	5.6	0.24			
day 112	5.3	5.2	0.16			
day 140	5.5	5.6	0.19			
day 175	6.0	5.7	0.17			
day 210	6.1	5.7	0.19			

^1^Treatments were as follows (*n* = 3 pens/treatment): 1) offered free-choice access to a corn-based diet with no MWDGS (CON) or 2) offered free-choice access to a diet with 40% MWDGS (DST; DM basis). Bulls (initial BW = 316 ± 29 kg, initial age = 240 ± 6 d) were offered treatments for 140 d and, then, switched to a free-choice, common, low-energy diet for an additional 70 d.

^2^Abbreviations are defined as treatment effect (Trt) and treatment × time effect (Trt × Time).

^3^Hoof evaluation was quantified using the American Angus Association’s simple foot scoring system, which characterizes cattle for two traits: foot angle and claw set. Both scores are ranked on a 1–9 scoring system with 5 being ideal.

Treatment × time and treatment effects were not detected (*P* ≥ 0.13; Table 5) for SC or SCORD. Furthermore, a time effect was detected (*P* < 0.01) for SC and SCORD, which both increased over time. Additionally, treatment × time and treatment effects were not detected (*P* ≥ 0.53; Table 6) for percentage of bulls that were pubertal or percentage of bulls with a satisfactory BSE. However, time effects were detected (*P* < 0.01) for percentage of bulls that were pubertal and percentage of bulls with a satisfactory BSE; more bulls became pubertal and had satisfactory BSE over time.

**Table 5.** Effects of offering growing bulls a diet with 40% MWDGS (DM basis) on SC and SCORD over time

	Treatment^1^			*P*-value^2^		
Item	CON	DST	SEM	Trt	Time	Trt × Time
SC^3^, cm				0.35	<0.01	0.13
day 28	27.3	27.4	0.45			
day 56	30.7	31.1	0.45			
day 84	32.7	33.1	0.45			
day 112	36.2	37.0	0.45			
day 140	36.8	36.3	0.46			
day 175	36.8	37.6	0.46			
day 210	39.2	40.6	0.46			
SCORD^4^, cm				0.42	<0.01	0.18
day 28	18.0	19.1	1.00			
day 56	24.1	24.9	1.00			
day 84	24.0	24.8	1.02			
day 112	30.6	32.3	1.00			
day 140	32.4	32.6	1.02			
day 175	35.7	35.4	1.02			
day 210	33.3	36.2	1.02			

^1^Treatments were as follows (*n* = 3 pens/treatment): 1) offered free-choice access to a corn-based diet with no MWDGS (CON) or 2) offered free-choice access to a diet with 40% MWDGS (DST; DM basis). Bulls (initial BW = 316 ± 29 kg, initial age = 240 ± 6 d) were offered treatments for 140 d and, then, switched to a free-choice, common, low-energy diet for an additional 70 d.

^2^Abbreviations are defined as treatment effect (Trt) and treatment × time effect (Trt × Time).

^3^SC was measured in centimeters using a scrotal tape measure placed around the widest portion on the scrotum.

^4^SCORD was measured in centimeters using a scrotal tape measure placed around scrotum between the top of the testis and the body wall.

**Table 6.** Effects of offering growing bulls a diet with 40% MWDGS (DM basis) on the percentage of bulls that were pubertal and passed a BSE over time

	Treatment^1^		*P*-value^2^		
Item	CON	DST	Trt	Time	Trt × Time
Pubertal^3^, %			0.53	<0.01	0.81
day 1	0	0			
day 28	13.3	15.0			
day 56	33.3	15.0			
day 84	56.7	43.3			
day 112	71.7	51.7			
day 140	85.0	86.7			
day 175	100.0	93.3			
day 210	100.0	100.0			
BSE^4^, %			0.59	<0.01	0.98
day 1	0	0			
day 28	0	0			
day 56	0	0			
day 84	21.7	16.7			
day 112	36.7	23.3			
day 140	58.3	44.3			
day 175	56.7	73.3			
day 210	63.3	75.7			

^1^Treatments were as follows (*n* = 3 pens/treatment): 1) offered free-choice access to a corn-based diet with no MWDGS (CON) or 2) offered free-choice access to a diet with 40% MWDGS (DST; DM basis). Bulls (initial BW = 316 ± 29 kg, initial age = 240 ± 6 d) were offered treatments for 140 d and, then, switched to a free-choice, common, low-energy diet for an additional 70 d. Values are expressed as back-transformed (sin[y])^2^ means.

^2^Abbreviations are defined as treatment effect (Trt) and treatment × time effect (Trt × Time).

^3^Bulls were determined to have reached puberty when at least 50 million sperm with at least 10% progressive motility were present in the ejaculate according to Wolf et al. (1965).

^4^BSE according to Koziol and Armstrong (2018).

**Figure 3.**
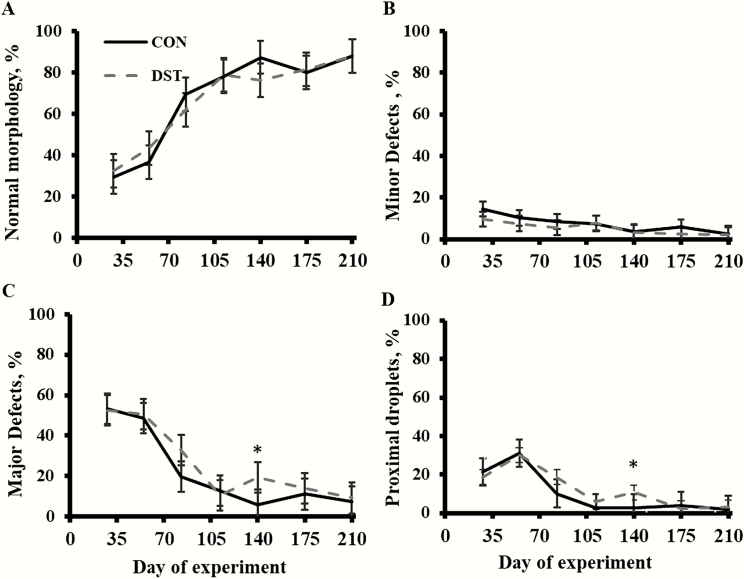
Effects of offering growing bulls a diet with 40% MWDGS (DM basis) on percentage of bull sperm with percentage of sperm with normal morphology (A), percentage of minor sperm defects (B), percentage of major sperm defects (C), and percentage of sperm with proximal droplets (D) in bull semen over time. Treatments were as follows (*n* = 3 pens/treatment): 1) offered free-choice access to a corn-based diet with no MWDGS (CON) or 2) offered free-choice access to a diet with 40% MWDGS (DST; DM basis). Bulls (initial BW = 316 ± 29 kg, initial age = 240 ± 6 d) were offered treatments for 140 d and, then, switched to a free-choice, common, low-energy diet for an additional 70 d. Significance of slice *P*-values are represented as: *P* ≤ 0.05 defined by *, and tendencies from 0.05 < *P* ≤ 0.10 are defined as †. Values are expressed as back-transformed (log_e_) means. Vertical bars represent the SEM of the untransformed means. Treatment × time and treatment effects were not detected (*P* ≥ 0.12) for sperm with normal morphology or minor sperm defects. A treatment × time effect was detected (*P* < 0.02) for major sperm defects. There was a tendency (*P* = 0.09) for a treatment × time effect for percentage of sperm with proximal droplets. A time effect was detected (*P* < 0.01) for sperm with normal morphology, minor sperm defects, major sperm defects, and sperm with proximal droplets.

Treatment × time and treatment effects were not detected (*P* ≥ 0.12; Figure 3) for percentage of sperm with normal morphology or percentage of minor sperm defects. A treatment × time effect was detected (*P* < 0.02; Figure 3) for percentage of major sperm defects. Bulls fed DST had a greater (*P* < 0.01) percentage of major sperm defects on day 140 than control bulls. There was a tendency (*P* = 0.09; Figure 3) for a treatment × time effect for percentage of sperm with proximal droplets. Bulls fed DST had a greater (*P* = 0.01) percentage of sperm with proximal droplets on day 140 than CON bulls. A time effect was detected (*P* < 0.01) for percentage of sperm with normal morphology, percentage of minor sperm defects, percentage of major sperm defects, and percentage of sperm with proximal droplets; percentage of sperm with normal morphology increased over time, whereas percentage of minor sperm defects, percentage of major sperm defects, and percentage of sperm with proximal droplets decreased over time.

**Figure 4.**
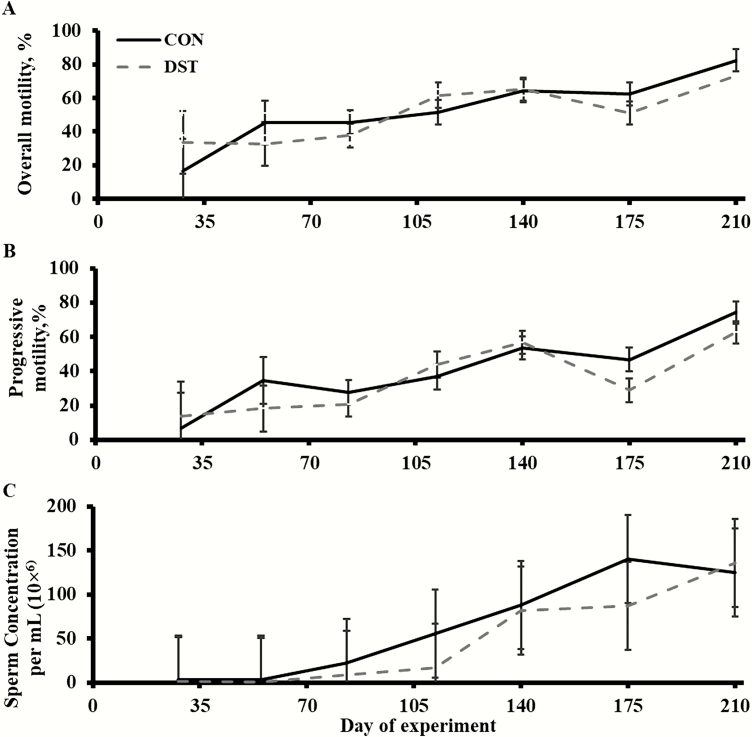
Effects of offering growing bulls a diet with 40% MWDGS (DM basis) on the overall sperm motility percentage (A), sperm progressive motility percentage (B), and sperm concentration (C) in bull semen. Treatments were as follows (*n* = 3 pens/treatment): 1) offered free-choice access to a corn-based diet with no MWDGS (CON) or 2) offered free-choice access to a diet with 40% MWDGS (DST; DM basis). Bulls (initial BW = 316 ± 29 kg, initial age = 240 ± 6 d) were offered treatments for 140 d and, then, switched to a free-choice, common, low-energy diet for an additional 70 d. Values for sperm concentration are expressed as back-transformed (*X*^0.25^) means. Vertical bars represent the SEM of the untransformed means. Treatment × time and treatment effects were not detected (*P* ≥ 0.40) for overall sperm motility percentage, sperm progressive motility percentage, and sperm concentration. However, a time effect was detected (*P* < 0.01) for overall sperm motility percentage, sperm progressive motility percentage, and sperm concentration.

Treatment × time and treatment effects were not detected (*P* ≥ 0.40; Figure 4) for overall motility percentage, progressive motility percentage, and sperm concentration. However, a time effect was detected (*P* < 0.01) for overall motility percentage, progressive motility percentage, and sperm concentration, which all increased over time.

## DISCUSSION

Bulls fed DST had improved BW at days 28 and 56, but this advantage was not observed at any of the remaining time points. Additionally, there were no differences in ADG, DMI, and G:F in the current study. Van Emon et al. (2013) and Crane et al. (2018) did not observe any effects on ram lamb final BW or ADG when comparing a control with no DDGS to increasing inclusions of DDGS. However, in a study by Lourenco et al. (2016), BW was greater for bulls fed a corn-based diet compared with a diet that contained 24.5% DDGS (DM basis). Conversely, Klopfenstein et al. (2008), in a meta-analysis, noted that, when wet distillers grains plus solubles (WDGS) were fed to finishing cattle, ADG and feed efficiency increased linearly with respect to the amount of WDGS added to the diet. However, DMI of those finishing animals decreased linearly in relation to the level of WDGS fed, but all levels (10%, 20%, and 30% WDGS; DM basis) were still greater than cattle on a control diet. In contrast, Richter et al. (2012) observed that steers supplemented with DDGS that was high in S (0.6%; DM basis) responded with decreased ADG compared with steers supplemented with DDGS that was low in S (0.2%; DM basis). In the current study, S was 0.24% of the DST diet (DM basis, Table 2). The National Academies of Science, Engineering, and Medicine (NASEM 2016) indicates potential S toxicity when S content is greater than 0.3% of the diet (DM basis) in concentrate-based diets. There were no observed cases of S toxicity in the current study.

**Table 2.** Chemical composition of diets fed to growing bulls during treatment period and following common, low-energy period

	CON transition steps^1^					DST transition steps					Low energy
Item	1	2	3	4	5	1	2	3	4	5	
DM, %	33.3	35.9	41.9	50.2	63.2	33.3	35.1	39.7	45.8	54.5	63.4
Chemical analysis		% of DM									
OM	92.4	92.7	93.1	93.9	94.4	92.4	92.4	92.0	92.5	92.7	90.7
CP	11.0	11.0	14.1	13.5	13.3	11.0	12.6	13.9	14.9	16.4	7.9
NDF	35.9	33.6	31.9	31.4	31.2	35.9	35.7	36.4	38.0	39.9	55.8
ADF	23.9	22.3	21.2	20.6	20.7	23.9	23.1	22.8	23.0	23.9	32.7
EE	2.8	3.0	3.1	3.2	3.2	2.8	3.6	4.2	4.9	5.5	1.7
mg/kg of diet DM											
Ca	10,996	10,804	11,019	9,674	9,119	10,996	10,876	12,865	11,592	11,109	10,577
P	2,865	2,966	2,849	2,715	2,635	2,865	3,469	3,883	4,251	4,673	1,555
S	1,534	1,518	1,555	1,475	1,430	1,534	1,768	1,948	2,118	2,323	1,108
Cu	15.2	14.9	12.3	11.7	11.3	15.2	15.3	14.3	14.1	14.0	11.1
Mn	81.2	79.8	63.8	59.4	56.5	81.2	80.9	76.2	72.9	71.0	113.2
Zn	71.3	71.2	60.5	60.8	61.5	71.3	74.7	78.7	82.6	86.7	50.0

OM, organic matter.

^1^Treatments were as follows (*n* = 3 pens/treatment): 1) offered free-choice access to a corn-based diet with no modified wet distillers grains plus solubles (MWDGS; CON) or 2) offered free-choice access to a diet with 40% MWDGS (DST; DM basis). Diets were fed to bulls (initial BW = 316 ± 29 kg, initial age = 240 ± 6 d) as follows: step 1 = days −35 to 1; step 2 = days 1 to 7; step 3 = days 8 to 14; step 4 = days 15 to 21; step 5 = days 22 to 140; low energy = days 141 to 210.

Bulls fed DST did have the composition of gain altered. The DST bulls had increased fat deposition. Larson et al. (1993) reported a linear increase in yield grade and marbling score when WDGS were fed at levels up to 40% (DM basis) to steers. Similarly, it was concluded by Klopfenstein et al. (2008) that linear increases in BF will be experienced with increasing levels of WDGS (10%, 20%, and 30%; DM basis) in the diets, while MS will be optimal at 30% inclusion (DM basis) of WDGS. In contrast, Lourenco et al. (2016) noted a decrease in ultrasound BF and RF of growing bulls fed a diet that included 25% DDGS (DM basis) compared with a corn-based control. In that same study, they noted no differences in MS or rib eye area.

Diet type and energy density have been known to affect the hoof quality and foot score of cattle (Lean et al., 2013). In the current study, there were no treatment differences in claw set score or foot angle score. However, over time, both scores changed. Foot angle initially got more upright, which is likely due to bulls transitioning to concrete floors covered in mats, followed by scores increasing and becoming more relaxed in angle. This change is expected as bulls mature and get to a heavier BW. Bull claw set became more curled over time, which is expected of animals on high-energy diets (Lean et al., 2013).

During the period when the bull is transitioned to a lower-energy diet, excessive BF is shed to reach an optimum BCS for the breeding season. This change in dietary energy level was implemented to replicate producer practices, as well as to provide an opportunity for reproductive convalescence (not necessarily performance convalescence). In this case, bulls quickly adjusted to the low-energy (**LE**) diet. Since all of the bulls were receiving the same diet for the final 70 d of the study, treatment differences in BW or ADG were not expected.

It is generally understood that breeding age bulls must have a minimum SC between 28 and 30 cm depending on breed (Wolf et al., 1965; Spitzer and Hopkins, 1997). Because of the relationship between puberty and SC, Koziol and Armstrong (2018) recommend a minimum scrotal circumference of 30 cm in all yearling bulls used for breeding. All bulls in the current study had an SC of 32 cm or greater on day 140, which suggests that all bulls were acceptable. Comparable gains for DST and CON bulls would indicate that net energy for growth was similar and, probably, the reason for no treatment effect on SC or SCORD. Crane et al. (2018) reported a linear decrease for ram SC when feeding increasing levels (0%, 15%, 30%, and 45%; DM basis) of DDGS. Previous work by Coulter and Kozub (1984) reported that feeding bulls increasing energy in their diets resulted in increased testicular size. Similarly, Martin et al. (1994) noted an increase in scrotal circumference when rams were fed the high-protein and high-energy rations compared to the rams fed the low-energy and low-protein rations. Hötzel et al. (1998) also observed an increase in SC throughout the study in rams fed to have an increased rate of gain. There were no differences in the percentage of bulls passing a BSE. On day 140 (bull age = 387 d ± 6), the percentage of bulls passing a BSE for CON and DST was 58% and 44%, respectively.

Bulls fed DST had an increase in percentage of major sperm defects on day 140 and this was a result of DST bulls having an increase in the percentage of sperm with proximal droplets. Proximal droplets are considered a major sperm defect. Barth and Waldner (2002) noted that it is not uncommon to find a higher percentage of sperm with proximal droplets in semen from yearling bulls. These defects typically resolve with age and a reduction in proximal droplet percentage would be expected at BSE conducted closer to time of sexual maturity (Barth and Waldner, 2002). No other nutritional cause for proximal droplets has been noted in the literature. Furthermore, the only study that has looked at feeding distillers grains with solubles and its impact on sperm morphology was performed by Crane et al. (2018), which actually noted no negative impacts on the morphology of spermatozoa and, for some traits, improved morphology when feeding increasing levels (0%, 15%, 30%, and 45%; DM basis) of DDGS. Based on the lack of major morphological defects, authors concluded that a total diet S of 0.4% (at 45% inclusion of DDGS; DM basis) was not leading to mineral deficiencies in vivo (Crane et al., 2018). This dietary S value is still greater than that of the current study where S was 0.23% of the DST diet (DM basis). In the current study, there was a time effect for percentage of normal sperm. As bulls increased in age and maturity, the percentage of normal sperm increased. Means for percentage of sperm with normal morphology would have met the minimal standards according to Koziol and Armstrong (2018) from day 112 to 210.

Overall motility percentage, progressive motility percentage, and sperm concentration were not affected by offering a diet with 40% MWDGS (DM basis). The previously mentioned study by Crane et al. (2018) actually noted a linear increase in spermatozoa concentration as DDGS concentration increased (0%, 15%, 30%, and 45%; DM basis) in the diets. However, in a similar study by Van Emon et al. (2013), they observed a linear decrease in spermatozoa concentrations in response to increasing levels of DDGS (0%, 15%, and 30%; DM basis) in the diet. Van Emon et al. (2013) indicated inadequate utilization of selenium and copper as a possible explanation for the observed reduction in spermatozoa concentration. According to the NASEM (2016), high-dietary levels of S can alter selenium and copper utilization and absorption. Excessive S intake can be toxic and could cause decreased performance and possibly cause PEM and death (Gould, 1998). The NASEM (2016) states that the maximum level of S tolerated in concentrate diets is 0.3% (DM basis). In the study by Van Emon et al. (2013), S was 0.5% (DM basis) when DDGS was included at 30% of the diet (DM basis), whereas Crane et al. (2018) noted S was 0.4% (DM basis) when DDGS was included at 45% of the diet (DM basis). In the current study, when MWDGS was included at 40% of the diet (DM basis), S was 0.23% of the diet (DM basis).

In conclusion, offering growing bulls a diet with 40% MWDGS (DM basis) resulted in heavier BW at days 28 and 56, increased RF at day 84, and increased BF and increases in both major sperm defects and sperm with proximal droplets at day 140. However, after 70 d on the common low-energy diet, there were no carryover effects on any growth, composition, hoof, or reproductive measure, except for a tendency for BF.
